# Strengthening support mechanisms for Accredited Social Health Activists in order to improve home-based newborn care in Uttar Pradesh, India

**DOI:** 10.1186/1753-6561-6-S5-O33

**Published:** 2012-09-28

**Authors:** Dharmendra S Panwar, Vandana Naidu, Emily Das, Shalini Verma, Abrar Ahmed Khan

**Affiliations:** 1 IntraHealth International, New Delhi, India

## Introduction

Accredited Social Health Activists (ASHAs) are female frontline health workers that provide critical antenatal and postnatal support to mothers and newborns for over five million deliveries in Uttar Pradesh every year. Performance reviews of ASHAs showed that even after initial training, the quality and the numbers of home visits made by ASHA were inadequate. ASHAs lacked interpersonal communication and counseling skills to effectively negotiate behavior change for home-based newborn care. The health system lacked mechanisms for continued learning and periodic upgrading of their skills and knowledge. Post-training, ASHAs were not being adequately supervised.

## Methods

The *Vistaar* project implemented by the IntraHealth Inc. worked with district health officials in five districts of Uttar Pradesh to create an alternative arrangement within the existing system for ongoing capacity building and supportive supervision of ASHAs. The monthly meetings of ASHAs were restructured, leading to more meetings but with smaller number of ASHAs (30-50 ASHAs at a time) on fixed days.

Structured content was developed for two-hour capacity building sessions covering IPC skills, delivering critical newborn care messages, use of job aids, planning home visits and organizing community meetings.

Facilitators at block level were identified from the health teams and were trained in the use of participatory methods and facilitation skills. They were assigned to facilitate capacity-building sessions during ASHA monthly meetings covering nearly 10,000 ASHAs every month. Auxiliary Nurse Midwives (ANMs) were trained on supportive supervision and were guided to enhance interactions with ASHAs including review of their performance and problem solving.

Technical Resource Groups were developed in districts for planning, implementation, and monitoring of the performance management support to ASHAs and ANMs. An end of project evaluation was carried out by an external agency.

## Results

ASHAs made home visits to 40% of all recently delivered women. Nearly 90% of recently delivered women reported first newborn checkups conducted by doctors and ANMs at the time of delivery at the healthcare facility. Compared to project baseline, a significant increase was reported in the second newborn care visit (20.6% to 60.3%) and the third visit (8.1% to 39.9%) by ASHAs. Recently delivered women were able to recall newborn care messages given by ASHAs during antenatal care visits including, initiating immediate breastfeeding within one hour of birth, exclusive breastfeeding up to six months, newborn immunizations, and benefits of colostrum feeding. Messaging on keeping newborns warm, delaying bathing for seven days and not applying anything to the cord need further improvement.

Breastfeeding within one hour of birth improved from 10% at baseline to 27.4% in the endline. Colostrum feeding showed a 22% improvement over the baseline to reach 79%. Capacity building sessions have been held in 93% of ASHA monthly meetings. ASHA attendance has improved significantly at 74 % in March 2012 compared to 48% in September 2009.

## Discussion

Ongoing capacity-building to periodically upgrade knowledge and skills of ASHAs is critical to building their confidence and translating technical knowledge into practice during home visits. Increased contacts and improved message delivery by ASHAs contribute to change in newborn care practices. Monthly meetings with about 30 to 50 ASHAs at a time provide an effective forum for ongoing capacity building and supportive supervision. Training facilitators in participatory methods and facilitation skills helps to use experience sharing by ASHAs for problem solving and peer learning. Providing supervisory skills and tools to ANMs can address essential support needs of ASHAs. Furthermore, interaction between ANMs and ASHAs during Village Health Nutrition Days provides an opportunity for one-on-one engagement and supervisory support. Working collaboratively with the government to utilize existing opportunities and restructure existing forums contributes to easier buy-in and system strengthening for sustaining the interventions beyond the project duration. It minimizes the cost of the effort mainly limiting it to the cost of staff time.

## Competing interests

Authors declare that they have no conflict of interest.

## Funding statement

This study was funded by the United States Agency for International Development Cooperative Agreement (Grant No. 386-A-00-06-00162-00).

